# The Clinical Value of ^*18*^F-FDG-PET in Autoimmune Encephalitis Associated With LGI1 Antibody

**DOI:** 10.3389/fneur.2020.00418

**Published:** 2020-06-05

**Authors:** Xiao Liu, Wei Shan, Xiaobin Zhao, Jiechuan Ren, Guoping Ren, Chao Chen, Weixiong Shi, Ruijuan Lv, Zhimei Li, Yaou Liu, Lin Ai, Qun Wang

**Affiliations:** ^1^Department of Neurology, Beijing Tiantan Hospital, Capital Medical University, Beijing, China; ^2^Beijing Institute for Brain Disorders, Beijing, China; ^3^China National Clinical Research Center for Neurological Diseases, Beijing, China; ^4^Department of Nuclear Medicine, Beijing Tiantan Hospital, Capital Medical University, Beijing, China; ^5^Department of Radiology, Beijing Tiantan Hospital, Capital Medical University, Beijing, China

**Keywords:** LGI1, ^18^F-FDG-PET, FBDS, basal ganglia, medial temporal lobe

## Abstract

**Purpose:** The metabolic patterns of ^18^F-fluoro-2-deoxy-*d*-glucose positron emission tomography (^18^F-FDG-PET) in autoimmune encephalitis associated with leucine-rich glioma-inactivated 1 antibody (LGI1 AE) are still unclear. We performed a cohort study to investigate the clinical metabolic characteristics and diagnostic value based on ^18^F-FDG-PET in patients with LGI1 AE.

**Materials and Methods:** A total of 34 patients including 18 patients (53%) in the acute phase and 16 patients (47%) in the chronic phase who were diagnosed with LGI1 AE were retrospectively analyzed from October 2014 to June 2018 at the Department of Neurology in Beijing Tiantan Hospital, the Capital Medical University. The clinical data were collected by searching through electronic medical records.

**Results:** The initial ^18^F-FDG-PET scan indicated a significant abnormal metabolic pattern in 31 LGI1 AE patients (91%), whereas only 20 patients (59%) showed an abnormal MRI signal (*P* < 0.05). The ^18^F-FDG-PET metabolic pattern was reversible after treatment; most of the patients showed an almost normal uptake of ^18^F-FDG-PET after discharge. Regarding the spatial distribution, the abnormal metabolic pattern in LGI1 AE subjects exhibiting hypermetabolism was specifically located in the basal ganglia (BG) and medial temporal lobe (MTL). BG hypermetabolism was observed in 28 subjects (82%), and 68% of patients showed MTL hypermetabolism. A total of 17 patients (50%) exhibited faciobrachial dystonic seizures (FBDS), and the remaining subjects showed non-FBDS symptoms (50 and 50%). BG-only hypermetabolism was detected in seven subjects in the FBDS subgroup (7/16) but in only one subject in the non-FBDS subgroup (1/15) (44 vs. 7%, *P* < 0.05).

**Conclusion:** ^18^F-FDG-PET imaging was more sensitive than MRI in the diagnosis of LGI1 AE. Isolated BG hypermetabolism was more frequently observed in subjects with FBDS, suggesting the potential involvement of the BG.

## Introduction

Autoimmune encephalitis (AE) associated with leucine-rich glioma-inactivated 1 (LGI1) antibody, described a decade ago, is a potentially treatable and recidivistic subtype of AE (1). It has been characterized as a subacute or rapidly progressive cognitive impairment accompanied by seizures, faciobrachial dystonic seizures (FBDS), and neuropsychiatric symptoms but rarely cancer (2). FBDS are highly specific and often appear as an initial symptom of LGI1 AE (3), which mainly presents with very frequent (medially 50 times per day) and brief involuntary movements of the ipsilateral face and limbs (usually <3 s for every episode) (4). However, whether FBDS should be treated as epileptic seizures is still controversial (5). Only certain studies have classified FBDS as epileptic-origin tonic seizures based on a detectable generalized electrodecremental event during the episode on ictal electroencephalogram (EEG) (6). However, other studies of LGI1 AE patients with FBDS have detected BG abnormalities on magnetic resonance imaging (MRI) that were not detected in subjects without FBDS, suggesting that FBDS might be a form of movement disorder (7). Hence, it is still difficult to confirm the origin or nature of FBDS.

For LGI1 AE patients, early diagnosis and treatment can prevent the development of the disease syndrome (8, 9). MRI is a preferred radiological modality in the early diagnosis of LGI1 AE, especially when antibody testing is negative or not available (10). Nevertheless, individual patients with LGI1 AE have been shown to exhibit negligible MRI findings, notably during the development of FBDS (4, 11, 12). Thus, it is necessary to find a novel or distinctive imaging pattern to expedite the early diagnosis of LGI1 AE (13). ^18^F-Fluoro-2-deoxy-*d*-glucose positron emission tomography (^18^F-FDG-PET) is a functional imaging modality for *in vivo* evaluation of the pathophysiology of the brain via application of ^18^F-FDG; it has been reported to reveal abnormal metabolism patterns in AE subjects, such as typical medial temporal lobe (MTL) hypermetabolism, especially in AE patients with a negative MRI, thus implying that ^18^F-FDG-PET has higher sensitivity than MRI in the diagnosis of AE subjects (14, 15). However, the ^18^F-FDG-PET pattern of patients with LGI1 AE is not well characterized or established. Regional basal ganglia (BG) or MTL hypermetabolism on ^18^F-FDG-PET has been observed in LGI1 AE patients (16–18). To date, only a limited number of isolated cases have been studied in subjects with LGI1 AE using ^18^F-FDG-PET.

Thus, we conducted a retrospective study and reviewed the ^18^F-FDG-PET data of 34 patients with a definite diagnosis of LGI1 AE based on symptoms, EEG, and LGI1 antibody testing. We evaluated the diagnostic value of ^18^F-FDG-PET in LGI1 AE subjects, especially those with unremarkable MRI alterations, and we also aimed to interpret the localization of FBDS by showing different metabolic abnormalities of ^18^F-FDG-PET in LGI1 AE patients with or without FBDS.

## Materials and Methods

### Standard Protocol, Approvals, and Patients' Consents

The study was approved by the Ethics Committee of the Beijing Tiantan Hospital, which was affiliated with the Capital Medical University of the People's Republic of China. The study was conducted in accordance with the Declaration of Helsinki, and all patients and controls provided informed consent for the use of their medical records.

### Study Participants

A total of 34 patients with LGI1 AE were retrospectively identified between October 2014 and June 2018 at the Department of Neurology in the Beijing Tiantan Hospital of the Capital Medical University. The inclusion criteria were based on representative clinical symptoms of LGI1 AE and the presence of positive LGI1 antibodies in the serum or cerebrospinal fluid (CSF). All included patients had undergone MRI and ^18^F-FDG-PET scans for neurological assessment during clinical evaluation. The demographic, clinical presentation, laboratory testing, EEG, and neuroimaging data were reviewed by searching the electronic medical records.

The 34 patients included 18 patients (53%) in the acute phase and 16 patients (47%) in the chronic phase when they take PET examination based on the previous definition of the acute phase (within 3 months) and chronic phase (over 3 months) in the diagnosis of AE (10).

The patients were divided into two subgroups based on the presence of FBDS, namely, FBDS and non-FBDS. We compared the ^18^F-FDG-PET findings in these two subgroups, analyzed the ^18^F-FDG-PET hypermetabolic states in the BG of the subjects, and then inferred the possible etiology or nature of FBDS.

In this study, we randomly selected additional 20 age- and gender-matched controls (14 men and 6 women; median age 62.5 years; range, 25–83 years) for the quantitative analysis of FDG-PET based on volume of interest (VOI). The inclusion criteria are the following: (1) no brain diseases, (2) no mental disorders reported in the medical records, (3) no other diseases that indicated the brain function had been affected, (4) no abnormalities reported by the neuroradiologist, (5) adjustment for gender and age and random pickup of the control subjects.

### Laboratory Detection

All patients underwent serum and CSF antibody detection, including *N*-methyl-d-aspartate receptor (NMDAR), LGI1, contactin-associated protein-2 (CASPR2), α-amino-3-hydroxy-5-methyl-4-isoxazolepropionic acid receptor (AMPAR), and γ-aminobutyric acid type B (GABAB). Serum and CSF samples were tested for the presence of LGI1 antibodies, using both cell-based assays (Euroimmun, Lübeck, Germany) and immunohistochemical analyses in the neuroimmunology laboratory of the Peking Union Medical College Hospital.

### ^18^F-FDG-PET Acquisition

^18^F-FDG-PET images were acquired using a PET/computed tomography (CT) scanner (Elite Discovery, GE HealthCare, Fairfield, Connecticut, USA). All patients (with a median age of 61 years, ranging from 31 to 78 years) fasted for at least 6 h, and blood glucose levels could not exceed 8 mmol/L. No patients received neuroleptic drugs to undergo FDG-PET. ^18^F-FDG was intravenously injected at a dose of 3.7–5.0 MBq/kg within 1 min, and subsequent uptakes required that patients be in a quiet resting status for 1 h prior to scanning in a dedicated room after ^18^F-FDG injection. First, a low-dose CT scan was performed, and the CT parameters for attenuation correction were 120 kV, pitch 0.984, automated tube current 60–180 mA, and slice thickness 3.75 mm. The PET scan was subsequently performed in 3D-TOF mode; for the LGI1 patients, a whole-body (including the brain region) FDG-PET scanning was acquired for approximately 30–35 min. The brain imaging data were reconstructed into trans-axial slices with a matrix size of 128 × 128 and a slice thickness of 3.3 mm, using an OSEM (ordered subset expectation maximization) algorithm.

### Voxel-Based Analysis of Statistical Parametric Mapping

For statistical parametric mapping (SPM) analysis, 22 age-matched, healthy volunteers served as control subjects. PET data were analyzed by SPM8 software (Wellcome Department of Cognitive Neurology, University College, London, UK) running on Matlab 2014b (MathWorks Inc., Sherborn, MA, USA). First, PET images were co-registered with the SPM template T1-weighted MR. Co-registered PET images were then spatially normalized into a common Montreal Neurological Institute (MNI) atlas anatomical space following a 12-parameter affine transformation and non-linear transformations, yielding images composed of 2 mm × 2 mm × 2 mm voxels. Third, normalized images were smoothed using an isotropic Gaussian kernel to increase the signal-to-noise ratio. Subsequently, preprocessed PET image values were corrected to a mean value of 50 ml/dl/min by “proportional scaling” to reduce individual variation. A two-sample *t*-test, based on the specified 22 age- and gender-matched controls, was applied between included patient PET data and the control group and to best reduce the impact of age and gender. The regions were considered significant at a corrected level of *P* < 0.01 for a minimum cluster size of 100 contiguous voxels (8 mm^3^ per voxel size). We purposefully chose a low threshold to detect any regions in which *V*_T_/*f*_P_ was more significant in patients.

### Data Analysis Based on VOI of Standardized Uptake Max Value

For these reported data, the region of interest (ROI) refers to the structure in 2D space (e.g., on a slice), and VOI refers to the structure in 3D space (i.e., a combination of ROIs of adjacent slices in two dimensions was labeled as a VOI). The value for radioactivity in each structure for each participant is the maximum of all pixels in all ROIs assayed for that structure, yielding a VOI. In our study, we selected three consecutive axial sections to define a certain volume (parameters included length, width, and height) to obtain the SUVmax (AW work station, GE HealthCare, USA). We calculated the SUVmax in three VOIs on FDG-PET based on the visual analysis, including the frontal cortex, BG, and MTL. The size of VOIs we selected was fixed, the center coordinate referred to the central location of VOIs, and they were individualized. One of the representative parameters of the individual was as follows: For the frontal cortex, the size of the volume was 25 ^*^ 20 ^*^ 9.9 mm, and the center coordinate of the volume was 72, 135, 30. For the BG, it was 40 ^*^ 30 ^*^ 9.9 mm, and the center coordinate was 109, 110, 23. For the MTL, the size of volume was 40 ^*^ 25 ^*^ 9.9 mm, and the center coordinate of volume was 112, 103, 18 (Supplementary Figure 1). The steps of quantitative comparison were as follows. (1) In the brain, the cubic VOIs were placed manually in the lesion. The SUVmax in the BG, MTL, and the frontal cortex was measured by three experienced neuroradiologists and nuclear imaging specialists (Lin Ai, Xiaobin Zhao, and Yaou Liu). All three specialists were blinded from clinical information of the conditions of either patients or controls, and in case of obvious discordance in their initial evaluations, an informed consensus statement was reached. The kappa coefficient of the three specialists was 0.88. (2) For normalization of SUVmax in the BG and MTL, the respective uptake values were divided by the values measured in the frontal VOI, which was chosen as the internal reference, as it was not expected to be altered in activity in LGI1 AE. Finally, we made a statistical comparison between 20 age- and gender-matched controls and 34 patients. (3) Receiver operating characteristic (ROC) curve analyses of normalized SUVmax in the BG and MTL for controls and patients were performed, and accordingly, optimal threshold values were set to 1.8 for BG and 1.3 for MTL.

### Literature Review

An extensive literature search was performed for the terms “positron emission tomography” and “encephalitis” from January 2007 to October 2018. The reported results were reviewed and summarized. The primary search identified 112 publications on PubMed, and any studies and case reports that showed abnormal metabolism on brain PET in patients with AE were included. The subtypes of AE included NMDAR, LGI1, CASPR2, and GABAB. Ultimately, 19 studies and case series were reviewed (Table 4).

### Statistical Analysis

Continuous variables with a normal distribution are presented as the mean ± standard deviation, and non-normal variables are expressed as the median (interquartile range, IQR). Continuous variables were compared using the *t*-test or non-parametric Mann–Whitney *U*-test. Categorical variables were compared and analyzed by Fisher's exact test. A two-tailed *P* < 0.05 was considered statistically significant. SPSS Statistics 23.0 software package for Windows (IBM Corp., Armonk, NY) was used for statistical analyses.

## Results

### Clinical Characteristics

A total of 34 patients (24 men; a median age of 61 years, IQR 54–65 years old; age range, 31–78 years) with LGI1 AE were identified, and their clinical characteristics were reviewed (Table 1). A total of 33 patients (97%) exhibited epileptic seizures, and the remaining one subject presented only with FBDS. Complex partial seizures (CPS) and generalized tonic–clonic seizures (GTCS) were the two main patterns of epileptic seizures. A total of 17 patients (50%) presented with distinctive FBDS, which involved both the arm and ipsilateral face (71%), and only 24% of patients experienced disturbances of awareness. Other symptoms were memory loss (88%), psychiatric disorders (depression, 9%; hallucinations, 26%, disorder of behavior, 24%), somnipathy (50%), and hallucinations (26%). Only one patient (3%) exhibited colorectal adenoma, and the remaining 33 patients did not present with a tumor after a median follow-up of 1.55 years (range, 0.3–4 years).

**Table 1:** Summary of the clinical characteristics of patients with LGI1 AE (*n* = 34).

**Characteristics**	**Values**
Age, year, median (IQR, range)	61 (54–65, 31–78)
Sex, male, *n* (%)	24 (71)
Clinical symptoms, *n* (%)	
FBDS	17 (50)
Seizures (except FBDS)	33 (97)
Memory loss	30 (88)
Psychiatric symptoms	20 (59)
Depression	3 (9)
Hallucinations	9 (26)
Disorder of behavior	8 (24)
Somnipathy	17 (50)
Hyponatremia, *n* (%)^a^	22 (65)
Tumors, *n* (%)	1 (3)
LGI1 antibody positive, *n* (%)	34 (100)
Only in serum	2 (6%)
Only in CSF	2 (6%)
Both in serum and CSF	30 (88%)
CSF abnormalities, *n* (%)^b^	10 (29)
EEG abnormalities, *n* (%)	
Total^c^	25 (74)
Ictal (FBDS)^d^	0 (0)
MRI abnormalities, *n* (%)	
Total	20 (59)
Only MTL lesion	18 (53)
Only BG lesion	1 (3)
Both BG and MTL lesions	1 (3)
^18^F-FDG-PET abnormalities, *n* (%)	
Total	31 (91)
Only MTL lesion	3 (9)
Only BG lesion	8 (23)
Both BG and MTL lesions	20 (59)
Immunotherapy, *n* (%)	34 (100)
Relapse, *n* (%)	8 (24)

*LGI1, leucine-rich glioma-inactivated 1; AE, autoimmune encephalitis; IQR, interquartile range; FBDS, faciobrachial dystonic seizures; CSF, cerebrospinal fluid; EEG, electroencephalogram; MRI, magnetic resonance imaging; MTL, medial temporal lobe; BG, basal ganglia; ^18^F-FDG-PET, ^18^F-fluoro-2-deoxy-d-glucose positron emission tomography*.

a *Normal range 135–145 mmol/L*.

b *CSF leukocyte count of >5/μl or protein levels of >45 mg/dl were considered abnormal*.

c *Temporal area slow waves or epileptiform discharges were considered abnormal*.

d *Relevant discharges on video EEG during the ictal phase of FBDS were considered abnormal*.

### Laboratory Testing

All 34 patients (100%) were positive for antibodies against the LGI1 protein (Table 1). LGI1 antibodies were commonly detectable in serum (94%) and CSF (94%), although two patients (6%) had positive LGI1 antibodies only in the CSF and in the serum (one patient each). Hyponatremia was reported for 22 patients (65%) (Table 1). A total of 10 out of 34 patients (29%) exhibited CSF pleocytosis (median, 7 white blood cells/μl; range, 6–16) and an increased protein concentration (median, 65 mg/dl; range, 49–73 mg/dl).

### Brain Image Review of LGI1 AE Patients

A total of 20 patients (59%) exhibited T2-weighted image (T2WI) or fluid-attenuated inversion recovery (FLAIR) hyperintensity on MRI. No subjects exhibited abnormal signals on T1-weighted image (T1WI). Increased signals were noted only in the MTL for 18 out of 34 patients (53%). A single subject (3%) showed isolated BG hyperintensity, and the remaining one (3%) had hyperintensity in both the MTL and BG.

A total of 31 patients (91%) showed an abnormal metabolism as determined by ^18^F-FDG-PET, and all of them (100%) presented with pure hypermetabolism. MTL and BG were two distinct metabolic targets in LGI1 AE patients (Figures 2A,C). Three patients (9%) exhibited increased glucose metabolism only in the MTL, whereas eight patients (23%) demonstrated BG-only hypermetabolism. The remaining 20 patients (59%) had increased metabolism in both the MTL and BG brain regions. Therefore, BG hypermetabolism was observed in 28 subjects (82%), and 68% of patients showed MTL hypermetabolism.

**Figure 1:**
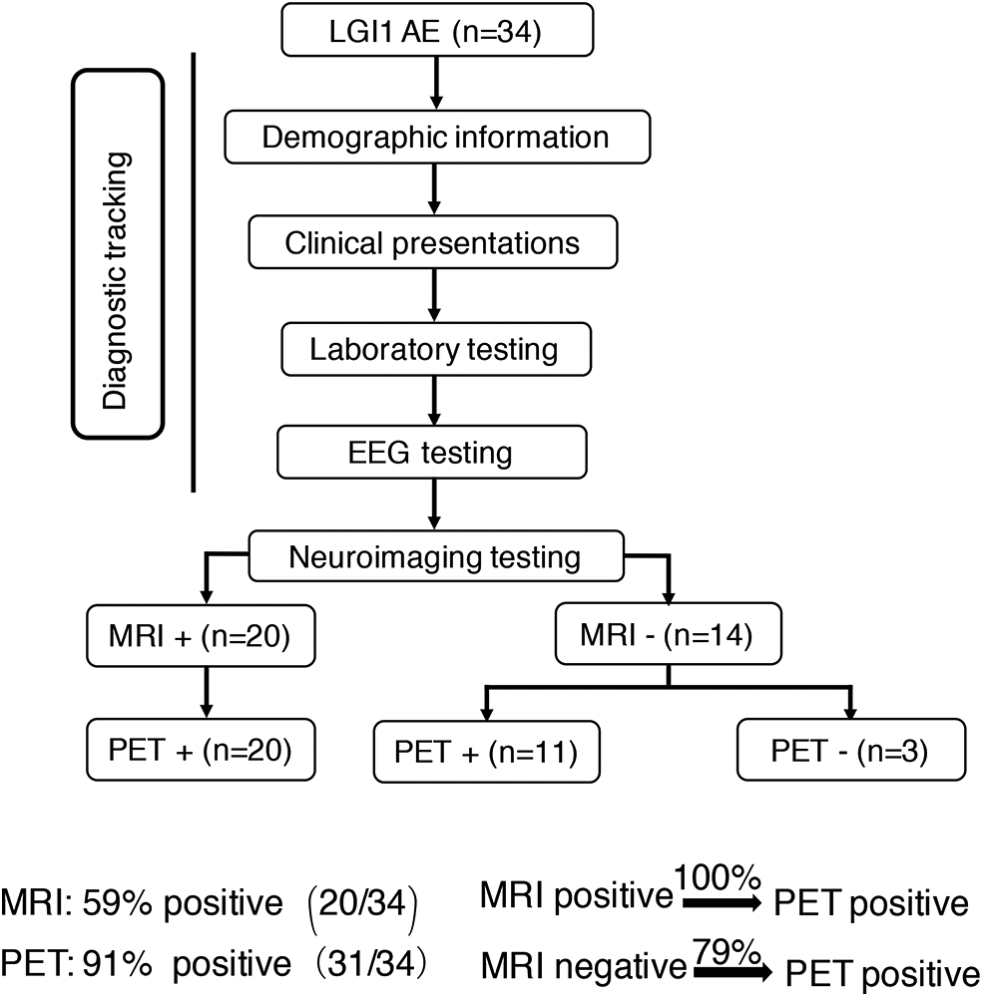
Neuroimaging testing in the diagnostic tracking of LGI1 AE. Neuroimaging testing plays an essential role in the diagnosis of LGI1 AE. The sensitivity of ^18^F-FDG-PET was higher than that of MRI; the ^18^F-FDG-PET scan was always positive when the MRI was positive, but when the MRI scan was negative for the diagnosis of LGI1 AE, 79% of the patients were still positive on the ^18^F-FDG-PET scan. LGI1, leucine-rich glioma-inactivated 1; AE, autoimmune encephalitis; EEG, electroencephalogram; MRI, magnetic resonance imaging; PET, ^18^F-fluoro-2-deoxy-*d*-glucose positron emission tomography; +, positive; –, negative.

**Figure 2:**
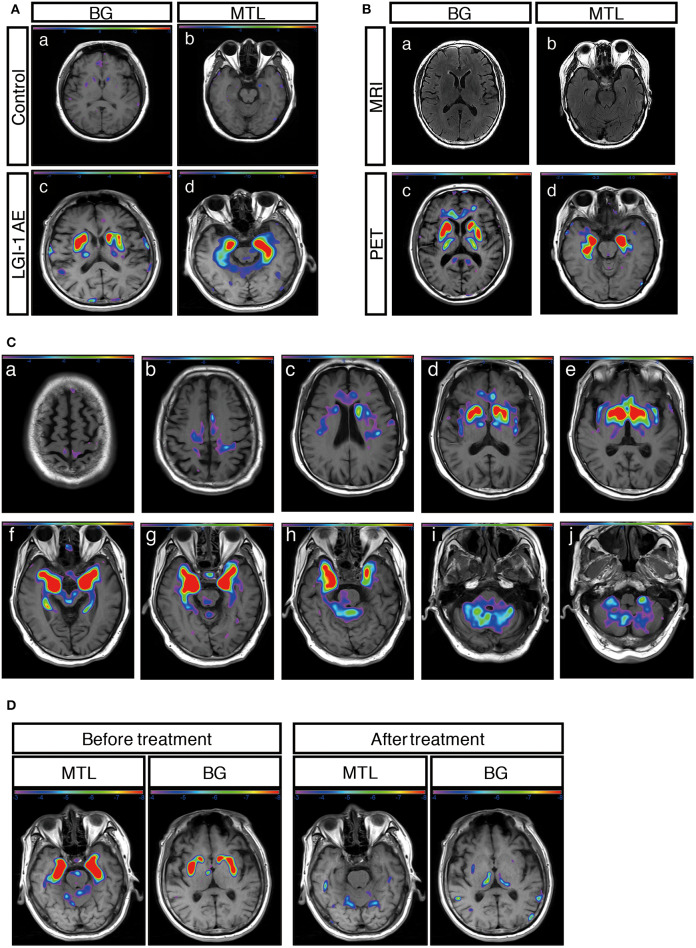
^18^F-FDG-PET metabolic pattern in patients with LGI1 AE. **(A)** Typical BG and MTL hypermetabolism based on ^18^F-FDG-PET in patients with LGI1 AE. Age-matched control patients with colorectal cancer in the absence of central nervous system lesions indicating healthy metabolism in the BG (a) and MTL (b). Representative increased metabolism in the BG (c) and MTL (d) in a patient with LGI1 AE. **(B)** MRI-negative and PET-positive metabolic patterns in LGI1 AE subjects. The axial fluid-attenuated inversion recovery image indicates a normal signal in the BG (a) and MTL (b), whereas ^18^F-FDG-PET reveals hypermetabolism in the identical location from the same patient with LGI1 AE (c,d). **(C)** The brain sequences in ^18^F-FDG-PET with LGI1 AE patients. Total brain ^18^F-FDG-PET mapping indicates the BG and MTL as two distinctive targets in LGI1 AE patients (d–h). **(D)** The reversible metabolic pattern in LGI1 AE. ^18^F-FDG-PET indicates increased ^18^F-FDG uptake in the BG and MTL. Furthermore, the ^18^F-FDG PET scan shows markedly decreased ^18^F-FDG uptake in the BG and MTL during the 3-month follow-up period. LGI1, leucine-rich glioma-inactivated 1; AE, autoimmune encephalitis; MRI, magnetic resonance imaging; PET, ^18^F-fluoro-2-deoxy-*d*-glucose positron emission tomography; MTL, medial temporal lobe; BG, basal ganglia.

Concerning the diagnosis of LGI1 AE, a comparison was made between the MRI and ^18^F-FDG-PET methods (Figure 1). Our diagnostic tracking showed that the sensitivity of ^18^F-FDG-PET was apparently superior to that of MRI (91 vs. 59%, *P* < 0.05), but no significant difference was noted between the two imaging modalities in regard to the median time from onset to the initial scan (82.5 vs. 75 days, *P* > 0.05). ^18^F-FDG-PET exhibited 100% metabolic changes when the MRI scans were positive; 11 out of 14 patients (79%) had altered glucose metabolism as demonstrated by ^18^F-FDG-PET in the presence of normal or unremarkable MRI scans (Figure 2B), and seven of them (64%) had isolated BG hypermetabolism.

A total of 12 subjects (35%) received a follow-up by ^18^F-FDG-PET, and all of them showed markedly decreased or normal uptake of ^18^F-FDG compared with the initial degree of metabolism; follow-ups occurred 68 ± 10 days following clinical treatment (Figure 2D).

### ^18^F-FDG-PET Pattern Among Subtypes of LGI1 AE Patients

**Figure 3:**
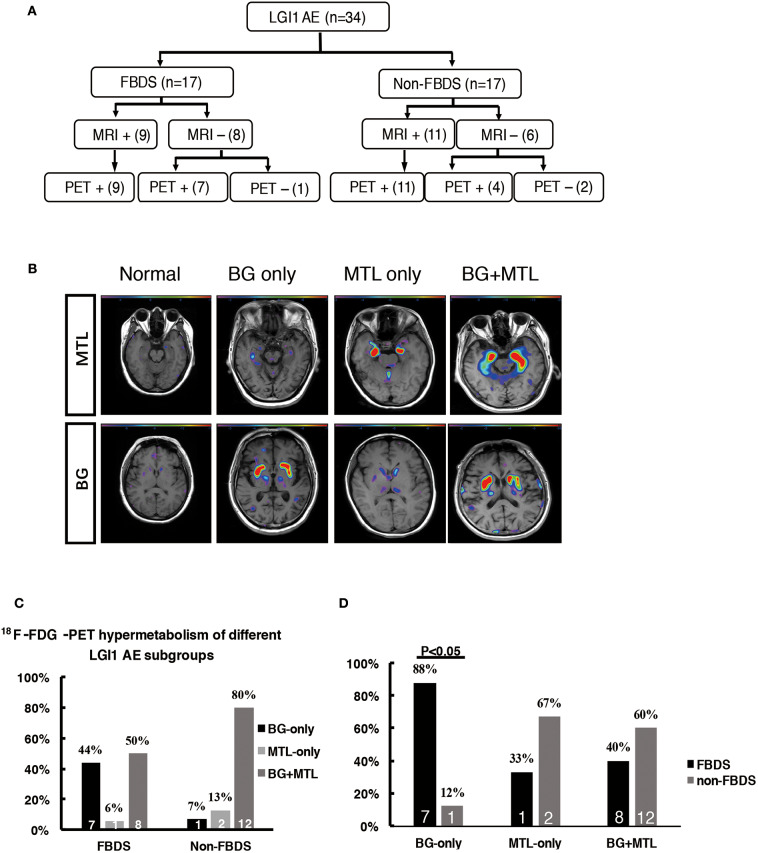
^18^F-FDG-PET hypermetabolism in LGI1 AE patients with FBDS. **(A)** Neuroimaging comparison of the subgroups of LGI1 AE. **(B)** Representative ^18^F-FDG-PET hypermetabolism among different subgroups after SPM (*P* < 0.01) analysis. **(C)** The comparison of the ^18^F-FDG-PET hypermetabolic pattern between FBDS and non-FBDS. In the FBDS group, the abnormal PET signal more often appeared in the BG-only group and the BG + MTL group. However, in the non-FBDS group, the abnormal PET signal significantly appeared in the BG + MTL group. **(D)** A comparison of the ^18^F-FDG-PET hypermetabolic pattern in FBDS and non-FBDS. In the BG-only group, the frequency of FBDS was higher than that of non-FBDS (*P* < 0.001). LGI1, leucine-rich glioma-inactivated 1; AE, autoimmune encephalitis; FBDS, faciobrachial dystonic seizures; MRI, magnetic resonance imaging; PET, ^18^F-fluoro-2-deoxy-*d*-glucose positron emission tomography; MTL, medial temporal lobe; BG, basal ganglia. +, positive; –, negative.

The patients were divided into two subgroups as follows: FBDS (*n* = 17) and non-FBDS (*n* = 17). The imaging data are summarized in Tables 2, 3. In the FBDS subgroup, ^18^F-FDG-PET was more sensitive than MRI (94 vs. 53%, *P* < 0.05). A similar result was noted in the non-FBDS subgroup, although this did not reach statistical significance (88 vs. 65%, *P* = 0.12). For the analysis of the MRI-negative patients, the sensitivity of ^18^F-FDG-PET in the FBDS subgroup was significantly higher than that of the subjects in the non-FBDS subgroup (50 vs. 29%, *P* < 0.05; Figure 3A).

**Table 2:** ^18^F-FDG-PET characteristics of LGI1 AE patients with FBDS (*n* = 17).

**Patient**	**FBDS**			**Time from onset to MRI (day)**	**MRI**	**Time from onset to ^**18**^F-FDG-PET (day)**	^****18****^**F-FDG-PET before treatment**		**Follow-up** ^****18****^**F-FDG-PET**	
	**Involvement**	**Loss of awareness**	**Ictal EEG**		**T2WI/FLAIR hyperintensity**		**Hypermetabolism**	**Hypometabolism**	**Time from treatment to follow-up (day)**	**Metabolic changes**
1	Arm, Face	No	–	425	MTL	143	BG, MTL	–	45	Decreased
2	Arm, Face	No	Normal	75	Normal	80	BG, MTL	–	–	–
3	Arm	No	–	130	Normal	13	Normal	–	–	–
4	Arm, Face, Leg	Yes	Normal	174	MTL	188	MTL	–	112	Decreased
5	Face	No	–	92	MTL	98	BG, MTL	–	–	–
6	Arm, Face	Yes	Normal	214	MTL	91	BG, MTL	–	142	Decreased
7	Arm, Face, Leg	Yes	–	100	Normal	50	BG	–	82	Decreased
8	Arm, Leg	No	–	14	BG	25	BG	–	–	–
9	Arm, face	No	Normal	28	MTL	21	BG, MTL	–	–	–
10	Arm, Face	No	Normal	305	Normal	302	BG	–	–	–
11	Arm, Face	Yes	Normal	34	BG, MTL	35	BG, MTL	–	98	Decreased
12	Arm, Face	No	–	95	Normal	332	BG	–	–	–
13	Arm, Face	No	Normal	297	Normal	45	BG	–	27	Decreased
14	Arm, Face	No	–	257	Normal	276	BG	–	–	–
15	Arm, Face	No	Normal	17	Normal	94	BG	–	–	–
16	Arm, Face	No	–	45	MTL	127	BG, MTL	–	53	Decreased
17	Arm, Face	No	–	12	MTL	17	BG, MTL	–	–	–

*^18^F-FDG-PET, ^18^F-fluoro-2-deoxy-d-glucose positron emission tomography; LGI1, leucine-rich glioma-inactivated 1; AE, autoimmune encephalitis; FBDS, faciobrachial dystonic seizures; EEG, electroencephalogram; MRI, magnetic resonance imaging; MTL, medial temporal lobe; BG, basal ganglia*.

**Table 3:** ^18^F-FDG-PET features of LGI1 AE patients with non-FBDS (*n* = 17).

**Patient**	**EEG**		**Time from onset to MRI (day)**	**T2WI/FLAIR hyperintensity**	**Time from onset to ^**18**^F-FDG-PET (day)**	^****18****^**F-FDG-PET before treatment**		**Follow-up** ^****18****^**F-FDG-PET**	
	**Ictal**	**Interictal**				**Hypermetabolism**	**Hypometabolism**	**Time from treatment to follow-up (day)**	**Metabolic changes**
1	–	TAD	12	MTL	11	MTL	–	–	–
2	Normal	Normal	190	MTL	372	BG, MTL	–	56	Decreased
3	TAD, FAD	TAD, FAD	798	MTL	797	BG, MTL	–	42	Decreased
4	Normal	Normal	34	MTL	50	BG, MTL	–	78	Decreased
5	–	Normal	75	MTL	92	MTL	–	–	–
6	Normal	FAD	94	Normal	91	BG, MTL	–	–	–
7	–	Normal	33	Normal	32	BG, MTL	–	–	–
8	TAD	TAD	4	Normal	43	Normal	–	–	–
9	TAD	Normal	87	MTL	130	BG, MTL	–	–	–
10	FAD	FAD, TAD	51	MTL	32	BG, MTL	–	–	–
11	–	Normal	12	MTL	19	BG, MTL	–	61	Decreased
12	–	FAD, TAD	148	MTL	185	BG, MTL	–	–	–
13	–	Normal	71	Normal	85	Normal	–	–	–
14	FAD, TAD	Normal	7	MTL	371	BG, MTL	–	25	Decreased
15	TAD	TAD	96	MTL	77	BG, MTL	–	–	–
16	TAD	TAD	67	Normal	64	BG, MTL	–	–	–
17	–	FAD, TAD	50	Normal	67	BG	–	–	–

*^18^F-FDG-PET, ^18^F-fluoro-2-deoxy-d-glucose positron emission tomography; LGI1, leucine-rich glioma inactivated-1; AE, autoimmune encephalitis; FBDS, faciobrachial dystonic seizures; EEG, electroencephalogram; MRI, magnetic resonance imaging; TAD, temporal area slow waves or epileptiform discharges; FAD, frontal area slow waves or epileptiform discharges; MTL, medial temporal lobe; BG, basal ganglia*.

A total of 31 patients (FBDS = 16, non-FBDS = 15) demonstrated increased metabolic changes as demonstrated by ^18^F-FDG-PET analysis. We classified the metabolic pattern of the patients into three types (BG only, MTL only, and BG + MTL) based on the location of the lesions in every subgroup (Figures 3B–D). BG-only hypermetabolism was detected in 7 out of 16 subjects (44%) with FBDS, but this pattern was detected in only one patient with non-FBDS (1/15) (44 vs. 7%, *P* < 0.05; Figure 3C). In the subgroup of BG only, a total of seven patients (88%) presented with FBDS, and one subject manifested with non-FBDS (*P* < 0.05; Figure 3D). No significant differences were noted between the two subgroups regarding the time from onset to initial ^18^F-FDG-PET scan (70.5 vs. 72 days, *P* = 0.53).

### Literature Review on ^18^F-FDG-PET Findings in Subtypes of AE

A total of 124 subjects with AE and PET scans were reviewed as shown in Table 4. For anti-NMDAR encephalitis, ^18^F-FDG-PET mainly presented with hypometabolism in the occipital (63%) and parietal (42%) areas, in the BG (33%) and the temporal lobe (29%) (14, 19–27). Patients with CASPR2 and GABAB showed no specific metabolic pattern due to the limitations regarding the number of cases (19, 30–32). However, abnormal metabolism in the BG and temporal lobe was observed in 77 and 62%, respectively, of subjects with LGI1 AE, and this pattern was relatively specific compared to the results in other subtypes of AE (4, 11, 17–19, 21, 28, 29).

**Table 4:** Literature review of ^18^F-FDG-PET pattern in subtypes of AE.

**AE subtypes**	**References**	**Patient cases**	^****18****^**F-FDG-PET findings**	
			**Hypermetabolism (*N*)**	**Hypometabolism (*N*)**
NMDAR	(19)	3	BG (1)	Thalamus (2)
NMDAR	(20)	6	Temporal (6), Cerebellum (3), Frontal (6)	Occipital (3)
NMDAR	(21)	6	Temporal (5)	Parietal (6), Cingulate gyrus (1)
NMDAR	(22)	6	BG (4), Frontal (1), Temporal (1)	BG (1), Frontal (5), Temporal (4), Parietal (3), Occipital (6)
NMDAR	(23)	8	BG (6), Frontal (4), Temporal (2)	Occipital (7)
NMDAR	(14)	4	BG (2), Cerebellum (2)	Temporal (1), Parietal (1), Occipital (2)
NMDAR	(24)	1	BG (1)	–
NMDAR	(25)	1	–	Parietal (1), Occipital (1)
NMDAR	(26)	8	BG (2), Cerebellum (2), Occipital (1)	BG (2), Frontal (4), Temporal (3), Parietal (5), Occipital (6)
NMDAR	(27)	5	–	Frontal (3), Temporal (2), Parietal (4), Occipital (5)
LGI1	(4)	8	BG (4), Temporal (4)	BG (2), Temporal (2)
LGI1	(19)	1	Temporal (1)	–
LGI1	(21)	4	BG (3), Cerebellum (3), Occipital (2)	Cingulate gyrus (4)
LGI1	(28)	1	BG (1)	–
LGI1	(29)	5	BG (5), Temporal (3), Frontal (5)	–
LGI1	(17)	1	BG (1), Temporal (1)	–
LGI1	(18)	1	BG (1), Temporal (1)	–
LGI1	(11)	10	BG (7), Temporal (7)	–
LGI1	This study	34	BG (28), Temporal (23), Cerebellum (1)	–
CASPR2	(19)	2	BG (1)	–
CASPR2	(30)	3	BG (1), Thalamus (1)	Temporal (1), Occipital (1)
GABAB	(31)	5	Temporal (2)	The whole cerebral cortex (1)
GABAB	(32)	1	Temporal (1)	–

*^18^F-FDG-PET, ^18^F-fluoro-2-deoxy-d-glucose positron emission tomography; AE, autoimmune encephalitis; NMDAR, N-methyl-d-aspartate receptor; LGI1, leucine-rich glioma inactivated-1; CASPR2, contactin-associated protein-2; GABAB, γ-aminobutyric acid type B; BG, basal ganglia*.

### Semiquantitative Analysis Based on the ^18^F-FDG-PET Normalized SUVmax Value

**Figure 4:**
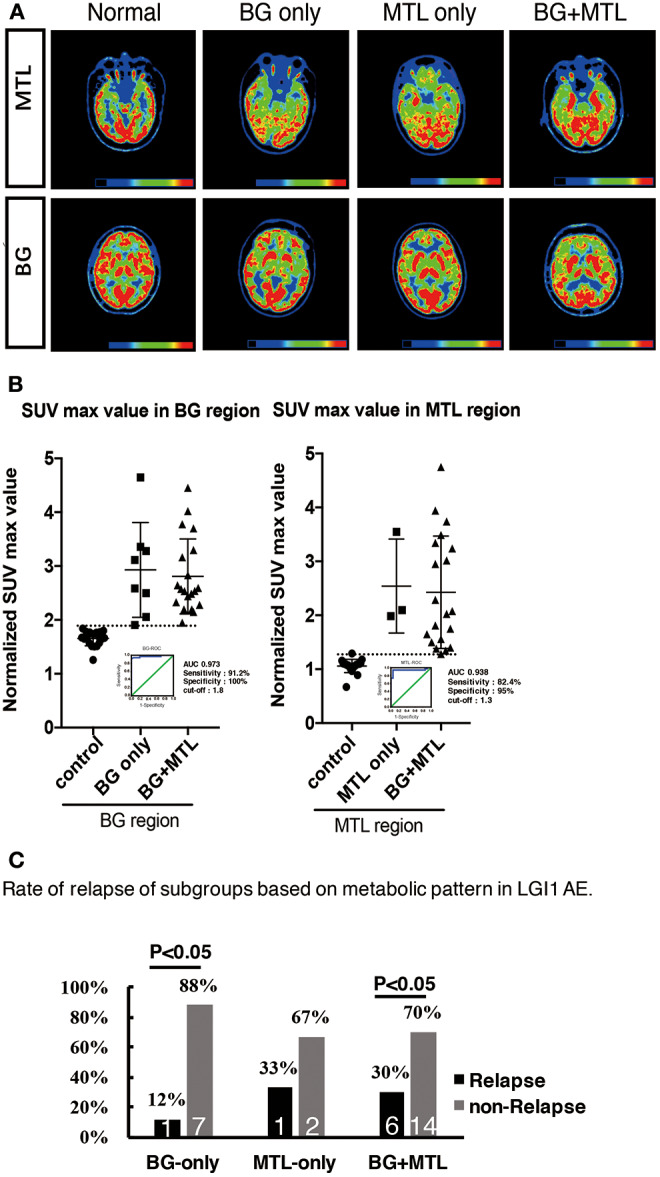
^18^F-FDG-PET hypermetabolism in eight patients with LGI1 AE recurrence. **(A)** Representations of the distribution of metabolic patterns without SPM analysis. **(B)** Normalized SUVmax value in different brain regions and the threshold of BG and MTL based on ROC. **(C)** The rate of relapse of the subgroups based on the metabolic pattern. The findings showed that patients presenting with BG-only or BG + MTL hypermetabolism had a lower rate of relapse (*P* < 0.05). LGI1, leucine-rich glioma-inactivated 1; AE, autoimmune encephalitis; ^18^F-FDG-PET, ^18^F-fluoro-2-deoxy-*d*-glucose positron emission tomography; BG, basal ganglia; MTL, medial temporal lobe.

To support the SPM voxel-based analysis results (*P* < 0.01) shown in our figures, we also performed a VOI standardized uptake max value (SUVmax) data analysis. We showed different raw metabolism patterns in different cases, such as normal, BG only, MTL only, and BG + MTL (Figure 4A), and quantified the normalized SUVmax value (Figure 4B). For the BG threshold, the ROC statistical results showed that the area under the curve (AUC) value was 0.973, that sensitivity was 91.2%, that specificity was 100%, and that the best cutoff value was 1.8. For the MTL threshold, the ROC analysis showed that the AUC value was 0.938, that sensitivity was 82.4%, that specificity was 95%, and that the best cutoff value was 1.3. Our semiquantitative analysis results showed that the normalized SUVmax value in the BG and MTL was higher than that in controls, which suggested that the metabolism of BG and MTL was indeed increased and further supported our SPM data.

### ^18^F-FDG-PET Characteristics of LGI1 AE Patients in Different Clinical Phases

**Figure 5:**
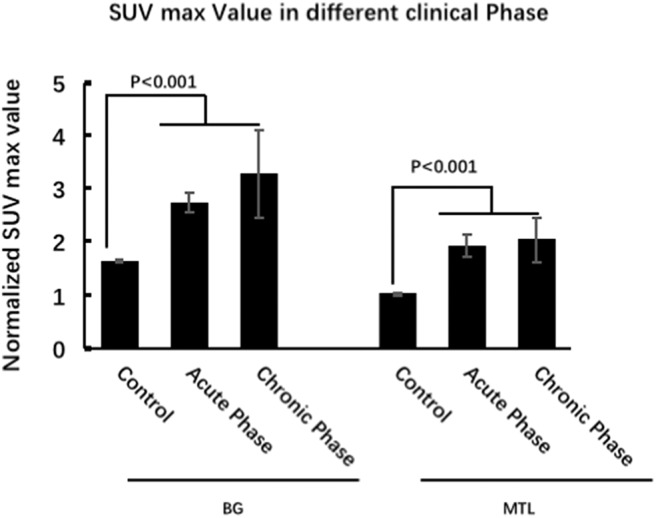
^18^F-FDG-PET characteristics of LGI1 AE patients in different clinical phases. FDG-PET hypermetabolism was specifically located in the BG and MTL, whether it was in the acute phase or chronic phase, and there is no statistical metabolic change between acute and chronic phases for BG and MTL. LGI1, leucine-rich glioma-inactivated 1; AE, autoimmune encephalitis; ^18^F-FDG-PET, ^18^F-fluoro-2-deoxy-*d*-glucose positron emission tomography; BG, basal ganglia; MTL, medial temporal lobe.

Eighteen patients (53%) were in the acute phase, and 16 patients (47%) were in the chronic phase at the time of PET. In the acute phase, patients with LGI1 AE on FDG-PET mainly presented with hypermetabolism in the BG (median normalized SUVmax = 2.5, IQR 2.2–3.4) and MTL (median normalized SUVmax = 1.5, IQR 1.3–3.2) compared with 20 normal subjects. BG (median normalized SUVmax = 2.3, IQR 2.1–3.1) and MTL (median normalized SUVmax = 1.4, IQR 1.3–2.1) on FDG-PET showed still a hypermetabolism in the chronic phase. FDG-PET hypermetabolism was specifically located in the BG and MTL, whether it was in the acute phase or chronic phase (*P* < 0.001). However, there was no statistical metabolic change between acute and chronic phases for BG and MTL (Figure 5).

### EEG Features

**Figure 6:**
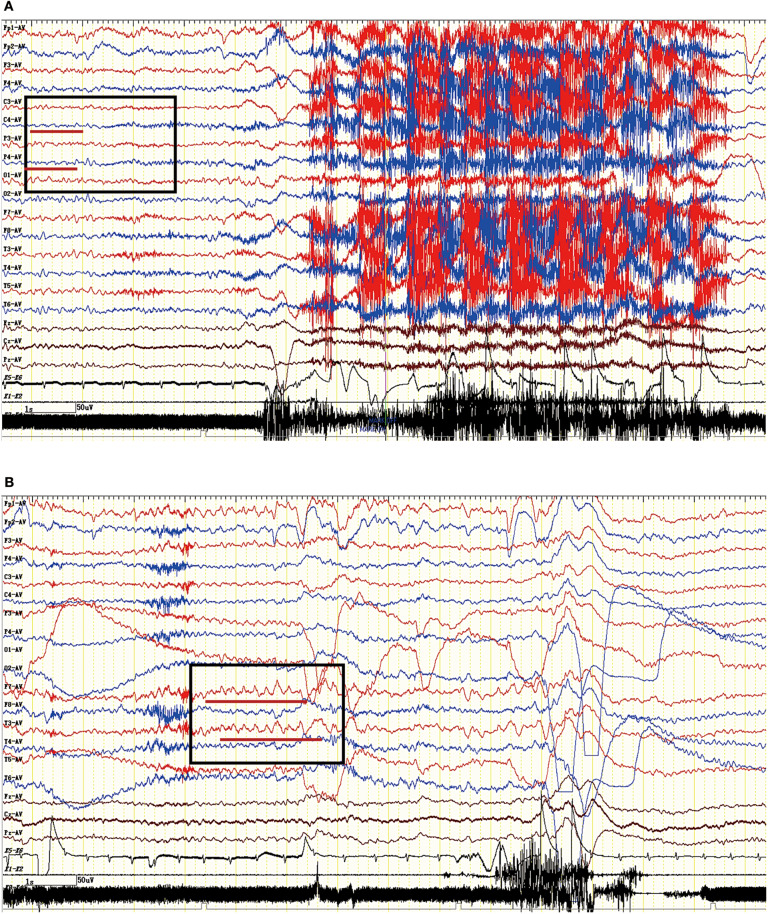
Ictal EEG pattern in an LGI1 AE patient with FBDS. **(A)** The ictal EEG of a 50-year-old woman with FBDS. No significant rhythm changes were noted except for artifacts of movements that were observed at the onset of the dystonic seizures. However, the EEG indicated low voltage in the right central and parietal areas before 5 s from onset (square frame). **(B)** The postictal EEG of the patient with FBDS. The EEG demonstrated rhythmic slow wave activity in the left temporal region (square frame) after the termination of FBDS, which was accompanied by hand automatism. LGI1, leucine-rich glioma-inactivated 1; AE, autoimmune encephalitis; FBDS, faciobrachial dystonic seizures; EEG, electroencephalogram.

The EEG characteristics of all subjects during the ictal and interictal phases were reviewed. A total of 25 patients (74%) experienced EEG abnormalities, which mainly included ictal or interictal EEG with slow wave activities and epileptic discharge in the temporal and frontal regions. However, no rhythmic discharges related to FBDS were noted except for artifacts of movements that were observed at the onset of dystonic seizures in eight patients with FBDS during the ictal phase. One subject with FBDS showed questionable low voltages in the right central and parietal areas around 5 s from onset (Figure 6A), and postictal EEG demonstrated rhythmic slow wave activity in the left temporal region after the termination of FBDS, which was accompanied by hand automatism (Figure 6B).

### Treatment and Follow-Up

All 34 patients (100%) were treated with first-line immunotherapy, including IV immunoglobulin (IVIG), IV methylprednisolone (IVMP), and oral steroids (for at least 6 months). A total of 26 patients (76%) were administered IVIG in combination with IVMP, whereas three patients (9%) used isolated IVIG and five patients (15%) received IVMP alone. Only one patient was administered azathioprine and mycophenolate mofetil (MMF) owing to the progression of the disease. Modified Rankin scores (mRS) were applied to evaluate the treatment response. Most patient conditions were improved following immunomodulatory therapies (mRS ≤ 2).

However, eight patients (24%) had a recurrence after a median follow-up of 1.55 years (range, 0.3–4 years). The distribution of metabolic patterns in the eight patients who relapsed is shown in Figure 4C. In the group with BG-only hypermetabolism, only one subject (12%) had a recurrence, and the remaining seven patients did not experience a relapse (*P* < 0.05). One patient (33%) who demonstrated MTL-only hypermetabolism experienced recurrence after a follow-up of 1 year. Six patients (30%) experienced a relapse, and 14 subjects did not relapse in the group with hypermetabolism in both the BG and MTL (*P* < 0.05). This showed that patients presenting with BG-only or BG + MTL hypermetabolism had a lower rate of relapse (*P* < 0.05).

## Discussion

In this study, we demonstrated that MRI plays a significant role in the diagnosis of anti-LGI1 encephalitis, whose main features are MTL or BG hyperintensities on T2WI/FLAIR. MRI was abnormal in 59% of subjects with LGI1 AE. We also described a specific metabolic pattern of ^18^F-FDG-PET among a cohort of subjects with anti-LGI1 encephalitis and further compared functional PET imaging with structural MRI regarding the diagnosis of LGI1 AE. The rate of abnormal brain metabolism based on ^18^F-FDG-PET imaging was 91%, and ^18^F-FDG-PET imaging was diagnostically more sensitive than MRI in patients with LGI1 AE, with 79% of subjects exhibiting altered glucose metabolism on ^18^F-FDG-PET in the absence of any abnormal MRI findings. The associated locations of the abnormal metabolism mainly included the BG and MTL, and the rate of abnormal findings in the BG and MTL was 82 and 68%, respectively. Our results suggest that ^18^F-FDG-PET abnormalities may support the evidence for a clinical diagnosis of subjects with anti-LGI1 encephalitis. In addition, this study introduces a novel ^18^F-FDG-PET pattern consisting of isolated striatal hypermetabolism in subjects with LGI1-mediated FBDS. Isolated striatal hypermetabolism was detected in 44% of subjects with FBDS but only in 7% of patients without FBDS. As a subject without FBDS exhibited isolated striatal hypermetabolism, we concluded that the BG might also be involved in the development of FBDS to a certain extent.

Brain glucose metabolism is closely associated with neuronal activity. Regional hypermetabolism may reflect exuberant neuronal activities induced by the inflammatory lesions of encephalitis. Furthermore, abnormalities in brain metabolic dysfunction usually change dynamically and currently precede structural changes. Our results primarily showed hypermetabolism or a healthy metabolism based on ^18^F-FDG-PET scans in LGI1 AE subjects at first hospitalization, which was potentially consistent with prior studies (4, 11). In addition, similar to previous studies (16, 17), ^18^F-FDG-PET indicated a reversible metabolic pattern in 12 patients who received follow-up. This observation supported the finding that ^18^F-FDG-PET exhibited an optimal correlation with disease severity and the hypothesis that it might be used to evaluate treatment response in LGI1 AE patients.

Our results mainly showed an abnormal metabolic pattern among LGI1 AE subjects in the BG and MTL. However, we also found abnormal hypermetabolism in the striatal, cerebellar, and cortex areas in some individual cases (data not shown) (21, 29). Furthermore, prior reports showed that BG and MTL hypermetabolism was also found in some individual cases in other subtypes of AE (20, 21). However, this observation was not specific to the metabolic pattern of anti-LGI1 encephalitis; our study reviewed ^18^F-FDG-PET patterns in AE and found that the BG and MTL were two distinctive targets in subjects with LGI1 AE compared to other subtypes of AE. Therefore, we may need more prospective studies to illustrate the metabolic pattern of other AE subtypes and to further evaluate the clinical value of striatal hypermetabolism for LGI1 AE subjects.

The origin of FBDS has been extensively debated, but no definite conclusions have been reached to date. Ictal EEG and neuroimaging examinations are the two main methods in current clinical use for explaining the etiology or localization of FBDS. On the one hand, ictal EEG mainly suggests an epileptic origin for FBDS, showing slow waves or epileptiform discharges in the temporal area during FBDS (4, 6, 33). On the other hand, neuroimaging examinations primarily suggest a BG localization, based on the evidence that the structural and functional changes in the striatum are easily detected in subjects with LGI1-associated FBDS (7, 18). Our study showed that pure BG hypermetabolism accounted for a higher proportion of FBDS, and it rarely appeared in subjects without FBDS in terms of functional ^18^F-FDG-PET. Furthermore, ictal EEG simultaneously indicated no rhythmic epileptic discharges during the ictal phase of FBDS. Hence, this study suggests that abnormalities of the BG might be involved in the etiology of FBDS and further supports the hypothesis that LGI1-associated FBDS is more likely a form of movement disorder rather than an epileptic disease in nature. However, some authors also hypothesize that FBDS might originate from a network dysfunction between the cortical and subcortical areas (28). Thus, future studies are crucial to provide more evidence to settle this controversy by correlating quantitative FDG metabolic uptake values in the BG with the severity of FBDS and further evaluating the existence of lateralized correlations between striatal metabolic changes and the physical aspects of FBDS.

The main limitations of this study are as follows. (1) The study is retrospective in nature: as not all subjects diagnosed with LGI1 AE during the observation period consented to performing an ^18^F-FDG-PET examination, a potential selection bias due to the small sample size may have been introduced. (2) Not all subtypes of AE were represented to be evaluated for an FDG pattern. (3) At the time point of the study, the diagnosis of LGI1 AE was mainly based on detection of antibodies, which might not necessarily match the final definite diagnosis in the further course of the disease. Therefore, our study was exploratory, and confirmatory tests should be taken with caution. Extensive prospective studies are required to verify the metabolism patterns of LGI1 AE, and a standardized ^18^F-FDG-PET protocol is also needed to meet the requirements for diagnosing LGI1-associated AE.

## Conclusion

^18^F-FDG-PET imaging was more sensitive than MRI in the diagnosis of anti-LGI1 encephalitis, and BG and MTL hypermetabolism are two distinctive targets for LGI1 AE compared to other subtypes of AE. Isolated BG hypermetabolism was more frequently observed in subjects with FBDS and potentially suggests the involvement of BG.

## Data Availability Statement

The datasets generated for this study are available on request to the corresponding author.

## Ethics Statement

The studies involving human participants were reviewed and approved by this study was approved by the Ethics committee of the Beijing Tiantan hospital that was affiliated to the Capital Medical University of the People's Republic of China. The patients/participants provided their written informed consent to participate in this study.

## Author Contributions

XL, WSha, and QW recruited, diagnosed, and assessed patients. XL, WSha, QW, XZ, JR, GR, CC, WShi, RL, ZL, and YL worked on the establishment of the separate databases. XL and WSha drafted a significant portion of the manuscript or figures. LA and QW reanalyzed and interpreted all final data. All authors contributed to the current version of the paper regarding conception or design, data analysis or editing, and read and approved the final manuscript.

## Conflict of Interest

The authors declare that the research was conducted in the absence of any commercial or financial relationships that could be construed as a potential conflict of interest.

## Supplementary Material

Supplementary Figure 1 The volume of quantitative ^18^F-FDG-PET based on VOI in one representative subject. For the frontal cortex, the size of the volume was 25 ^*^ 20 ^*^ 9.9mm, and the center coordinate of the volume was 72, 135, 30. For the MTL, the size of the volume was 40 ^*^ 25 ^*^ 9.9mm, and the center coordinate of the volume was 112, 103, 18. For the BG, it was 40 ^*^ 30 ^*^ 9.9mm, and the center coordinate was 109, 110, 23. ^18^F-FDG-PET, ^18^F-fluoro-2-deoxy-d-glucose positron emission tomography; VOI, volume of interest; MTL, medial temporal lobe; BG, basal ganglia.

## Abbreviations

^18^F-FDG-PET: ^18^F-Fluoro-2-deoxy-*d*-glucose positron emission tomography
AE: autoimmune encephalitis
AMPAR: α-amino-3-hydroxy-5-methyl-4-isoxazolepropionic acid receptor
BG: basal ganglia
CASPR2: contactin-associated protein-2
CSF: cerebrospinal fluid
CT: computed tomography
EEG: electroencephalogram
FBDS: faciobrachial dystonic seizures
GABAB: γ-aminobutyric acid type B
LGI1: leucine-rich glioma-inactivated 1
MTL: medial temporal lobe
MRI: magnetic resonance imaging
NMDAR: *N*-methyl-d-aspartate receptor
SPM: statistical parametric mapping
SUVmax: standardized uptake max value.
