# Supplementary figures and images for: The Clinical Value of 18F-FDG-PET in Autoimmune Encephalitis Associated With LGI1 Antibody
Source: Front Neurol. 2020 Jun 5;11:418. doi: 10.3389/fneur.2020.00418 (PMC7290050; doi:10.3389/fneur.2020.00418)

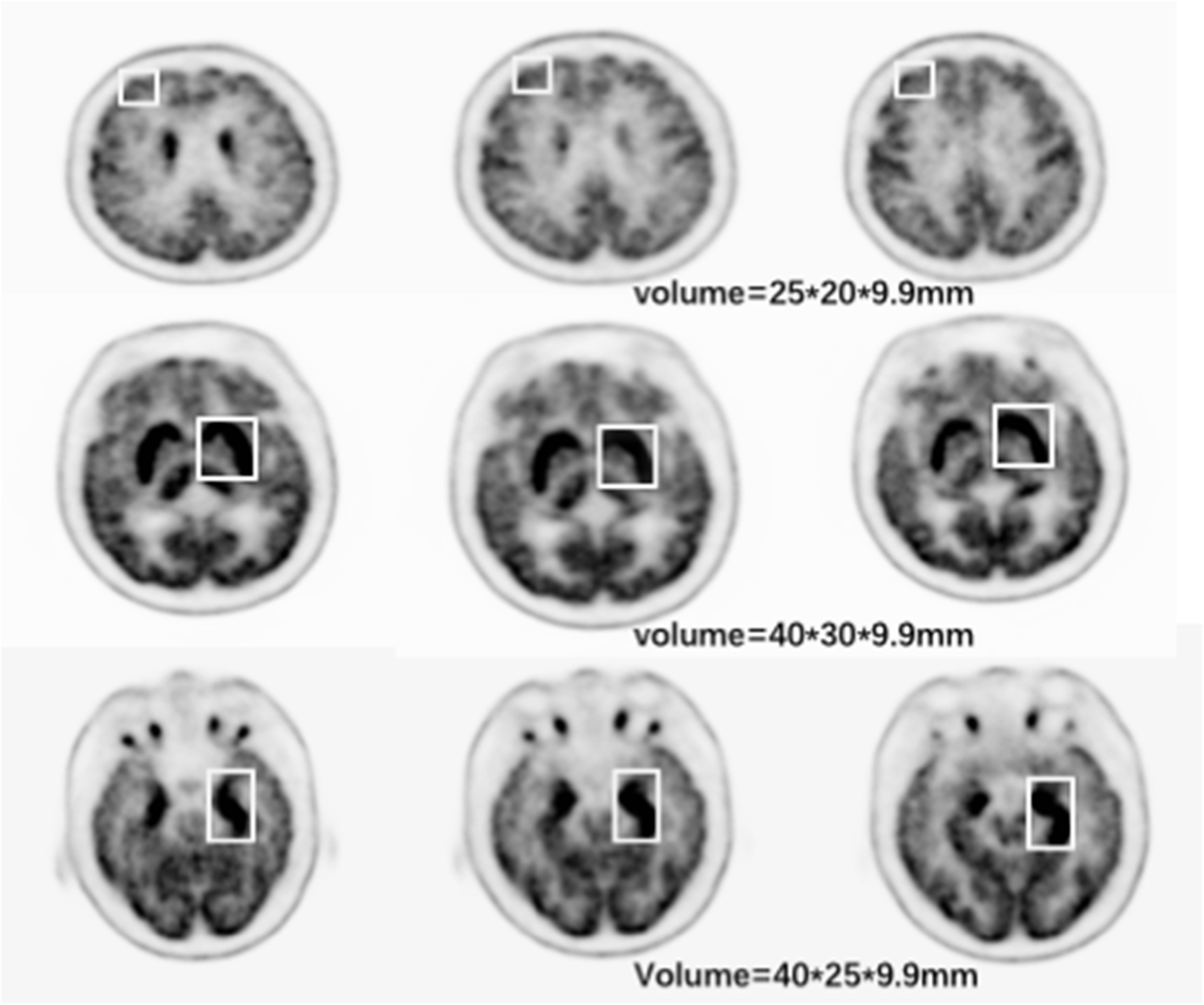

Supplement: Supplementary Figure 1 — The volume of quantitative 18F-FDG-PET based on VOI in one representative subject. For the frontal cortex, the size of the volume was 25 * 20 * 9.9mm, and the center coordinate of the volume was 72, 135, 30. For the MTL, the size of the volume was 40 * 25 * 9.9mm, and the center coordinate of the volume was 112, 103, 18. For the BG, it was 40 * 30 * 9.9mm, and the center coordinate was 109, 110, 23. 18F-FDG-PET, 18F-fluoro-2-deoxy-d-glucose positron emission tomography; VOI, volume of interest; MTL, medial temporal lobe; BG, basal ganglia. [file Image_1.TIF]
